# The Importance of Epigenetics in Diagnostics and Treatment of Major Depressive Disorder

**DOI:** 10.3390/jpm11030167

**Published:** 2021-03-01

**Authors:** Piotr Czarny, Katarzyna Białek, Sylwia Ziółkowska, Justyna Strycharz, Gabriela Barszczewska, Tomasz Sliwinski

**Affiliations:** 1 Department of Medical Biochemistry, Medical University of Lodz, 92-216 Lodz, Poland; piotr.czarny@umed.lodz.pl (P.C.); sylwia.ziolkowska@stud.umed.lodz.pl (S.Z.); justyna.strycharz@umed.lodz.pl (J.S.); 2 Laboratory of Medical Genetics, Faculty of Biology and Environmental Protection, University of Lodz, 90-236 Lodz, Poland; biaalek.k@gmail.com (K.B.); gabriela.barszczewska@edu.uni.lodz.pl (G.B.)

**Keywords:** depression, epigenetics, personalized medicine, histones, histone modifications, DNA methylation, microRNA, long non-coding RNA

## Abstract

Recent studies imply that there is a tight association between epigenetics and a molecular mechanism of major depressive disorder (MDD). Epigenetic modifications, i.e., DNA methylation, post-translational histone modification and interference of microRNA (miRNA) or long non-coding RNA (lncRNA), are able to influence the severity of the disease and the outcome of the therapy. This article summarizes the most recent literature data on this topic, i.e., usage of histone deacetylases as therapeutic agents with an antidepressant effect and miRNAs or lncRNAs as markers of depression. Due to the noteworthy potential of the role of epigenetics in MDD diagnostics and therapy, we have gathered the most relevant data in this area.

## 1. Introduction

Major depressive disorder (MDD) is a chronic, highly recurrent and clinically heterogeneous disease, also known simply as depression. It is characterized by high worldwide prevalence and increased risk for suicidal death [1,2]. MDD is no longer believed to be triggered by just one factor (e.g., biological, psychosocial, behavioral), but rather to occur due to interactions among several of them during one’s lifetime [2]. Considering genetics, the recent genome-wide association meta-analysis involving 135,458 MDD patients and 344,901 control subjects elucidated 44 independent risk variants, including 30 novel ones [3]. Nevertheless, the meta-analysis of five studies on twins estimated the heritability of the disease to account for 37% [4]. Therefore, it becomes clear that susceptibility to depression is not entirely explained by changes found in a DNA sequence. As the response to environmental stressors is diverse, stress exposure may evoke depression only in some individuals. Thus, it is not surprising that a current view on the etiology of depression is rather to perceive environmental and genetic factors as interacting with and modulating one another (G × E—gene–environment) [5]. Recently, a third plausible contributory factor has emerged called epigenetics. The latter denotes gene expression regulation via modifications not encoded/found in DNA nucleotide sequence, yet heritable and sensitive to environmental influence. The whole repertoire of epigenetic mechanisms is believed to enable a crosstalk among environment and genetics, thus respectively affecting the phenotype. A large body of evidence indicates not only that epigenetic factors may contribute to depression pathogenesis, but also have an impact on responses of individuals to pharmacotherapy. This appears to be an especially intriguing issue due to the fact that drug resistance is attributable to one-third of treated subjects [6,7]. Three well-known epigenetic mechanisms that sophisticatedly control the expression of genetic information are: (i) fifth carbon atom methylation of cytosine within its heterocyclic aromatic ring generating 5-methylcytosine (5 mC) [8] and its oxidation into 5-hydroxymethylcytosine (5 hmC) [9]; (ii) remodeling of chromatin via numerous covalent modifications, e.g., methylation, acetylation, phosphorylation, SUMOylation mainly in N-terminal tails of core histones [5]; (iii) actions mediated by non-coding RNAs, such as microRNAs (miRNAs) and long non-coding RNAs (lncRNAS) [10,11]. The above mechanisms stay in crosstalk among one another so as to coordinately change the phenotype. For instance, expression of miRNAs and lncRNAs is regulated via DNA methylation and remodeled chromatin, while DNA methylation marks (5-methylcytosines) are capable of recruiting modifying enzymes to histones [12]. Furthermore, more and more studies explore the impact of lncRNAs on miRNAs (e.g., acting as miRNA sponges) eliciting changes of target mRNAs in numerous diseases, including Alzheimer’s [13].

The exact mechanisms of molecular pathogenesis of depression are still not known, including the involvement of epigenetic ones. Thus, this review provides a summary of the state of the art of the association among epigenetics and pathogenesis and MDD therapy, while outlining promising directions for future research. We used Google Scholar and PubMed in order to review the most relevant papers focusing on epigenetic modifications in the context of diagnostics and treatment of major depressive disorder published until December 2020. We considered studies performed on animals as well as human subjects (in vivo, in vitro) along with clinical trials. Keywords applied were as follows: major depressive disorder, depression, depression biomarkers, depression diagnostics, microRNA, miRNA, lncRNA, DNA methylation, promoter methylation, histone modifications, histone acetylation, histone methylation, neuroplasticity, synaptic plasticity, neuroinflammation, depression therapy, antidepressants, nervous system, brain, blood, serum, plasma, cerebrospinal fluid.

## 2. DNA Methylation

### 2.1. DNA Methylation in Depression

One of the best-known heritable patterns of modifications that affect gene expression, which cannot be attributed to changes in the primary DNA sequence, is DNA methylation. Therefore, it is one of the most extensively studied epigenetic modifications in humans. This mechanism is mediated by a family of DNA methyltransferases (DNMTS), with DNMT3A/DNMT3B being responsible for de novo methylation and DNMT1 recognizing hemi-methylated DNA. The process occurs with the covalent addition of a methyl group almost exclusively at the cytosine base in 5′ position into the major groove of DNA leading to creation of 5-methylocytosine (5 mC). In mammals, DNA methylation predominantly occurs at palindromic sequence of cytosine–guanine dyads (CpG sites) within the promoter regions, yet, it is also found inside a coding sequence of the gene. The regulatory role of DNA methylation has been implicated in gene expression control, especially in transcription repression, due to reducing access of transcription factors to regulatory elements [8]. As approximately 3% of cytosines are methylated [14] in the human genome, it plays a pivotal role in many biological processes such as gene imprinting, chromosomal inactivation, cell differentiation, repetitive elements silencing [8]. An important discovery proved that 5 mC could be modified via oxidation into 5-hydroxymethylcytosine (5 hmC); it was reported in mammals in 2009 for the first time [9]. This process is called DNA demethylation and is catalyzed by ten-eleven translocation (TET) enzymes. Although it is present in many tissue types, 5 hmC is found to be especially abundant in neuronal cells of the brain. It is hypothesized that DNA methylation may play an important role in mediating stress effects. Moreover, methylation could establish gene silencing by its cooperation with histone modifications. Specifically, it is proposed that the mentioned interrelation may be mediated by proteins such as Zinc finger and BTB domain-containing protein 33 (ZBTB 33), methyl-CpG-binding protein 2 (MeCP2) and methyl-CpG-binding domain protein 1 (MBD1), which present methyl DNA binding activity and ability for recruitment of protein complex containing histone deacetylases (HDACs) and methyltransferases [15].

DNA methylation is among several epigenetic modifications especially interesting in the context of neuropsychiatric diseases. Environmental factors, which often influence disease trajectories, are able to modify methylation patterns [16,17]. Recent evidence indicates that alterations in DNA methylation, i.e., both hyper- and hypomethylation at the same loci as well as methylation variance, are frequently present in depressed individuals [18]. Methylation patterns are also characterized by great stability, therefore they could serve as possible biomarkers of the disease and even might be helpful in predicting treatment improvements [19]. Most of the knowledge accumulated to date on epigenetic mechanisms in depression comes from animal studies; however, these results have recently been confirmed in human research (Table 1). A particularly adequate way for studying epigenetic changes is to observe them in monozygotic twins, since they have nearly the same DNA sequence, but their methylation profiles could be different due to environmental or stochastic factors [20]. Interestingly, identification of differentially methylated probes (DMPs) and variably methylated probes (VMPs) evaluated discrepancies in DNA methylation between monozygotic twins and revealed a link between their psychopathological characteristic and those differences [21].

**Table 1:** Summary of the human studies assessing DNA methylation in depressive disorders.

Gene	Material	Model/Study Description	Antidepressant	Results	Study
*BDNF*	Buccal swab	French patients (age > 65) with depression	-	Significantly higher methylation of gene promoter.	[22]
	PBMC	Korean patients with MDD, before and after 12-week long antidepressant treatment	SSRIs, SNRIs, TCAs,	Better treatment response in patients with lower methylation of *BDNF* promoter.	[23]
	PBMC	Patients with MDD after antidepressant treatment	SSRIs, SNRIs	Decreased *BDNF* methylation after antidepressant treatment.	[24]
	PBMC	MDD patients before treatment with antidepressants and assessed outcomes at the study endpoint	-	Significantly lower methylation of *BDNF* in non-responders to antidepressant treatment.	[25]
	Buccal swab	Fifty-seven women with depressive symptoms during pregnancy and their infants	-	Prenatal depressive symptoms significantly decreased *BDNF* methylation in both male and female infants.	[26]
*SLC6A4*	PMBC and umbilical cord leukocytes (infants)	Women with depressive symptoms during pregnancy and their infants	-	Increased maternal depressed mood symptoms in the 2nd trimester were associated with the lower maternal and infant *SLC6A4* promoter methylation status.	[27]
	Peripheral blood	Patients with MDD and childhood trauma, during antidepressant treatment with SSRIs	SSRIs	Increased methylation of promoter after antidepressant treatment.	[28]
	Peripheral blood	Korean patients with MDD, before and after 12 week long antidepressant treatment	amitriptyline, bupropion, escitalopram, fluoxetine, imipramine, mirtazapine, paroxetine, sertraline, venlafaxine	Higher methylation of *SLC6A4* was associated with worst treatment response and worst depressive symptoms remission.	[29]
	Peripheral blood	84 monozygotic twin pairs with or without depressive symptoms	-	Intrapair DNA methylation variance was significantly correlated with intrapair differences in depressive symptoms.	[30]
	Peripheral blood	Caucasian MDD patients before and after antidepressant treatment with escitalopram	escitalopram	Hypomethylation of the promoter was associated with diminished response rate to escitalopram.	[31]
	Peripheral blood	Japanese patients with MDD before and after antidepressant treatment	paroxetine, fluvoxamine	Hypermethylation of the *SLC6A4* promoter was associated with better treatment response for paroxetine and fluvoxamine.	[32]
	Peripheral blood	Caucasian patients with MDD receiving antidepressant drugs	SSRIs, SNRIs, TCAs	Hypomethylation of *SLC6A4* predicted impaired antidepressant response.	[33]
*NR3C1*	Peripheral blood	MDD patients with childhood maltreatment and sexual abuse	-	Childhood sexual abuse, and maltreatment positively correlated with higher *NR3C1* methylation.	[34]
	Buccal swab	Fifty-seven women with depressive symptoms during pregnancy and their infants	-	Prenatal depressive symptoms significantly increased *NR3C*1 methylation in male infants.	[26]
*ZNF575*	Cord blood	Women with depressive symptoms during pregnancy, undergoing antidepressant treatment and their infants	SSRIs	Lower DNA methylation of *ZNF575* in newborns exposed to antidepressants in utero.	[35]
*PPFIA4*	Peripheral blood	Japanese patients with MDD receiving antidepressant drugs	paroxetine	Difference in promoter methylation was significantly correlated with treatment response to paroxetine.	[36]
*HS3ST1*	Peripheral blood	Japanese patients with MDD receiving antidepressant drugs	paroxetine	Difference in promoter methylation was significantly correlated with treatment response to paroxetine.	[36]
*IL-11*	Peripheral blood	Caucasian European patients with MDD receiving escitalopram or nortriptyline	escitalopram, nortriptyline	Lower methylation was associated with better treatment response; higher methylation was associated with better response to escitalopram, but with worse response to nortriptyline.	[37]
*TPH2*	Peripheral blood	Han Chinese patients with MDD	SSRIs, SNRIs, SARIs, antipsychotic drugs	Hypomethylation was associated with treatment response rate.	[38]

PBMC—Peripheral blood mononuclear cell, MDD—major depressive disorder, SSRI—serotonin selective reuptake inhibitor, SNRI—serotonin norepinephrine reuptake inhibitor, TCAs—tricyclic antidepressants.

#### 2.1.1. Brain-Derived Neurotrophic Factor (*BDNF*) Gene Methylation in Depression

The first genome-wide DNA methylation scan in depressed patients identified significant DNA methylation differences located in genes connected with neuronal growth and development [39]. It has been demonstrated that increased DNA methylation of the brain-derived neurotrophic factor (*BDNF*) gene, encoding the protein responsible for neuronal growth, differentiation and plasticity, is connected with depression in the human population [40]. It is established that BDNF could be involved in behavioral phenomena connected with depressive disorders as well as antidepressant treatment effectiveness. Higher methylation status of CpG in *BDNF* promoter region correlated with significantly lower synthesis of this factor in neurons [41]. Moreover, it has been confirmed that patients with depressive symptoms and those with suicidal ideation displayed increased methylation level of *BDNF* promoter [22,40,42]. Interestingly, during antidepressant treatment, a trend of decreasing *BDNF* methylation was observed [24].

Epigenetic modifications occurring in early life and even in utero may have long-term effects in later age. Together with environmental factors they could play an important role in development of neuropsychiatric diseases, including depression. For instance, maternal depressive symptoms during the prenatal period are frequently associated with hypomethylation of *BDNF* promoter in infants [26]. This statement is also confirmed by animal studies (Table 2), where methylation alterations were found in rats subjected to the animal model of childhood maltreatment, suggesting its association with stressful early-life experience [18,43]. Rodent studies indicated that caregiver ill-treatment caused an increased methylation of *BDNF* in the prefrontal cortex [43].

**Table 2:** Summary of the animal studies assessing DNA methylation in depressive disorders.

Gene	Material	Study Description	Antidepressant	Results	Study
*BDNF*	Prefrontal cortex and hippocampus	Rat model of infant maltreatment by a caregiver	-	Increased methylation of *BDNF* in the prefrontal cortex	[25]
	Hippocampus	Mouse model of post-stroke depression	Fluoxetine	Increased methylation of *BDNF* promoter in hippocampus as well as reduction in depressive behavior	[44]
*NR3C1*	hippocampus	Rat model: pups with increased frequency of licking, grooming and arched-back nursing by mother versus neglected rats	-	Offspring neglected by a mother showed changes in GR methylation levels	[23]
*THP1*	PBMC	Rats subjected to chronic mild stress procedure and administrated with venlafaxine	venlafaxine	Increased methylation status after antidepressant therapy	[45]
*GPX4*	PBMC	Rats subjected to chronic mild stress procedure and administrated with agomelatine	agomelatine	Decreased methylation status after antidepressant therapy	[46]
*NOS1*	Brain tissues	Rats subjected to chronic mild stress procedure and administrated with agomelatine	agomelatine	Decreased methylation status of gene promoter in amygdala and basal ganglia after antidepressant therapy	[46]
*GPX1*	Brain tissues	Rats subjected to chronic mild stress procedure and administrated with agomelatine	agomelatine	Decreased methylation status of gene promoter in hippocampus and hypothalamus after antidepressant therapy	[46]
*CAT*	Brain tissues	Rats subjected to chronic mild stress procedure and administrated with agomelatine	agomelatine	Decreased methylation status of gene promoter in cerebral cortex after antidepressant therapy	[46]

PBMC—Peripheral blood mononuclear cell, GR—Glucocorticoid receptor.

#### 2.1.2. *SLC6A4* Gene Methylation in Depression

A growing body of evidence suggests that the same trend is observed in the case of encoding for serotonin transporter *SLC6A4* gene, which has been especially well-studied in the context of serotonergic signaling in depression. Serotonin transporter plays a critical role in MDD, since it is responsible for serotonin turnover and level in synaptic cleft. Therefore, any disturbances of *SLC6A4* may contribute to depression pathogenesis, by causing dysregulation of the serotonergic system. Consistent with *BDNF,* maternal mood disturbances during a pregnancy period have been connected with decreased methylation status of *SLC6A4* in umbilical cord leukocytes of newborns [27]. However, a majority of recent evidence demonstrates an increase in DNA methylation of *SLC6A4* in patients with depressive disorders [8,28,30,42]. Moreover, promoter hypermethylation of the gene in peripheral blood was associated with a worse clinical outcome [29]. Studies using functional magnetic resonance imaging (fMRI) showed that higher methylation of *SLC6A4* promoter was connected with hippocampal volume and higher frontal-limbic response to negative stimulation in MDD subjects [47,48].

#### 2.1.3. *NR3C1* Gene Methylation in Depression

Among the potential candidate genes is the *NR3C1* gene, which encodes a glucocorticoid receptor (GR), an essential factor for sufficient hypothalamic–pituitary–adrenal (HPA) axis functioning. Precisely, cortisol, upon binding to GR, induces a cascade of stress-related responses to stress stimuli. Therefore, GR plays a pivotal role in termination of stress response by mediating negative feedback regulation of the HPA axis. This mechanism could be affected by variety of factors, and it is proposed that *NR3C1* methylation may be considered a feasible explanation for understanding HPA axis dysregulation, frequently occurring in depression [8]. Despite extensive research, results are inconsistent. On the one hand, there is some evidence for hypermethylation of the *NR3C1* gene in depressed females and their offspring [17]. On the other hand, recent research reported no association between its methylation and depression [8]. Moreover, animal studies indicated that offspring neglected by a mother showed changes in GR methylation levels in tissues from the hippocampus [49]. Confusingly, depressed patients who had experienced early sexual abuse and maltreatment, presented increased methylation of *NR3C1* promoter. Moreover, this methylation status was positively correlated with the severity of mistreatment [34].

### 2.2. DNA Methylation and Antidepressant Treatment

#### 2.2.1. Human Research

Epigenetic mechanisms are more often studied in the context of depression treatment. What is more, recent work focuses on biological predictors for drug response, identifying a modern approach to therapeutic strategies. This field is called pharmacogenetics (PGx) and provides opportunity for improved prognostication of response and tolerability of different medications. Growing research examines ways and mechanisms in which epigenetic marks predict treatment outcomes as a part of pharmacoepigenetics. There are some pieces of evidence suggesting that antidepressants deliver their effect at least partially through epigenetic modifications. An increasing amount of data indicate that epigenetic changes observed in patients with depressive disorders could act as predictors of antidepressant treatment response. Antidepressants may also have the ability to alter or even reverse epigenetic modifications, and therefore, it has been proposed that treatment response might be indicated by methylation status of some genes. In patients with post-stroke depression (PTSD), *NR3C1* methylation was associated with treatment outcome [50]. What is even more interesting, research conducted on pregnant women undergoing antidepressant treatment revealed that newborns exposed to antidepressants during pregnancy had lower DNA methylation of Zinc Finger Protein *575 (ZNF575)* gene sequence [35]. The clinical data suggest that *SLC6A4* promoter methylation status might be a possible marker for treatment response prognosis. Specifically, promoter hypomethylation of the gene has been linked with diminished response rate to escitalopram. It could have been caused by increased expression/activity of serotonin transporters, and therefore, insufficient availability of this neurotransmitter in the synaptic cleft [31]. This is in line with another study, which found that decreased methylation of *SLC6A4* promoter predicted diminished dimensional and categorical response to serotonergic antidepressant drugs [33]. Interestingly, hypermethylation of the *SLC6A4* promoter has been associated with improved treatment response for antidepressants, such as paroxetine and fluvoxamine [32] as well as escitalopram [31]. A large study investigated the role of *BDNF* methylation in MDD patients and its association with depression therapy. Lower methylation of *BDNF* promoter has been linked with antidepressant response rate, predicting non-responsiveness to multiple classes of antidepressants, including selective serotonin reuptake inhibitors (SSRIs), selective norepinephrine reuptake inhibitors (SNRIs), tricyclic antidepressants (TCAs), and monoamine oxidase inhibitors (MAOIs) [25]. In addition, *BDNF* methylation could be regulated by DNMT1 according to antidepressant response to paroxetine [51]. Similarly, methylation of PTPRF Interacting Protein Alpha 4 (*PPFIA4)* and Heparan Sulfate-Glucosamine 3-Sulfotransferase 1 *(HS3ST1)* genes was associated with therapeutic response to paroxetine [36]. Moreover, another potential predictor of treatment outcome might be an epigenetic status of interleukin 11 *(IL-11)* promoter, as it has been found that methylation of several CpG sites could be used to estimate whether an individual is likely to respond to antidepressants [37]. It is suggested that serotonin receptor 1A and 1B *(HTR1A/1B)* genes methylation could be involved in the pathogenesis of MDD and treatment response. It is indicated that hypomethylation of those genes and their interplay with stress stimuli might cause worsening of antidepressant treatment outcome. As mentioned, there are various mechanisms by which antidepressant drugs and their compounds could alter epigenetic marks. Some data suggest that amitriptyline and imipramine belonging to TCAs as well as SSRI (paroxetine), show an ability to reduce DNMT activity, and thus decrease DNA methylation [52]. Paroxetine has also been found to affect DNMT activity by changing its phosphorylation [51]. There are also studies reporting that methylation of the tryptophan hydroxylase 2 (*TPH2*) gene could predict response to antidepressants as well as interaction between hypomethylation and early-life stress may be associated with worse treatment outcome [38].

#### 2.2.2. Animal Models of Depression

Studies on animal models of depression are in line with results from human research. For instance, the methylation status of the *TPH1* in peripheral blood mononuclear cells (PBMCs) was found to be significantly increased after antidepressant therapy with venlafaxine [45]. There are also results indicating that agomelatine administration reduced methylation of Glutathione Peroxidase 4 (*GPX4)* promoter in PBMC as well as Nitric Oxide Synthase 1 *(NOS1)*, Glutathione Peroxidase 1 (*GPX1)* and Catalase *(CAT)* promoters in brain tissues of rats subjected to chronic mild stress [46]. Post-treatment observations showed that fluoxetine increased methylation of *BDNF* promoter in mice hippocampal tissues, which was also associated with a reduction in depressive behaviors [44]. Moreover, chronic imipramine distribution reversed methylation of a CpG within the *HTR1A* promoter in the prefrontal cortex and midbrain of mice [53], as well as corticotropin-releasing factor (*CRF)* gene in the mice hypothalamus [54], which were induced by stress stimuli. Furthermore, imipramine administration has been connected to decreasing stress-induced global DNA methylation and DNMT activity in the prefrontal cortex of rats [55]. It was also demonstrated that DNMT inhibitors enhanced the effect of conventional antidepressants and improved depressive-like behaviors in mice [56,57].

To conclude, the methylation rate of numerous genes may display predictive function for evaluating the effectiveness of antidepressant treatment. However, while exploring DNA methylation marks as biomarkers, it is important to design experiments that assess and account for a variety of potential confounders, including socioeconomic and demographic factors.

## 3. Histone Modifications

Histone modifications, more precisely, post-translational modifications (PTMs), and their functions are tightly related to gene expression control. There is a hypothesis assuming that the sum of these modifications, the so-called histone code, influences the expression level of targeted genes [58]. In the last decade, a lot of research has been focused on the association between the PTMs and neuropsychiatric diseases, and a growing number of researchers have noticed how important and evident this relationship is. In the case of depression studies, most attention has been paid to acetylation and methylation of histones.

**Figure 1:**
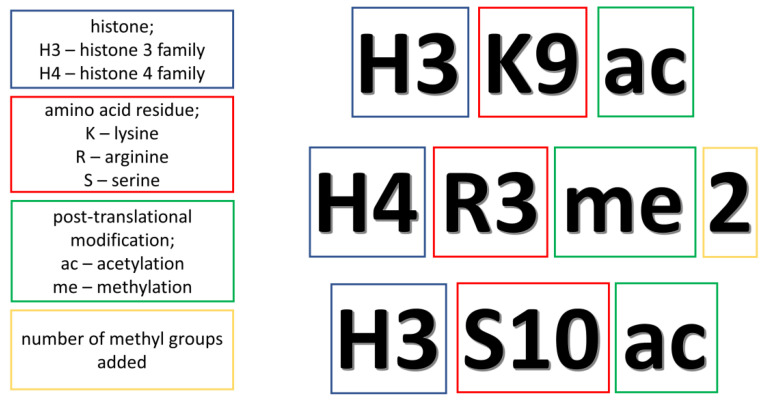
The explanation of abbreviations related to histone modifications.

Due to the inverse action of acetylation and deacetylation, they are able to control the chromatin condensation, and by this regulate gene expression. The former is catalyzed by histone acetylases (HATs), and the latter by histone deacetylases (HDACs). The effects of their presence are upregulation and downregulation of gene expression, respectively. Whereas, in the case of methylation, the expression is regulated by histone methyltransferases (HMTs) and histone demethylases (HDMs) and their action depends on the site of the methylated residue as well as on the degree of methylation [20]. For example, histone H3 lysine 27 trimethylation (H3K27me3) decreases expression, while H3K4me2/3 activates transcription (Figure 1) [59]. Apart from these two modifications, there are also some studies exploring the crotonylation that occurs in individuals with MDD [60]. Histone crotonylation is based on adding the short-chain acyl groups to amino acids (aa) and is performed through the transfer of crotonyl groups by crotonyl-CoA. This modification mostly appears in colon and brain and results in the activation of transcription [61].

The most frequent modifications discovered in depression are acetylation and methylation at the lysine residues, especially these of histone 3 or 4 [58]. These changes are remarked both in animal models of the disease and in patients with MDD. Due to this fact, these epigenetic modifications should be considered as the diagnostic markers of depression. However, this is not the only area where PTMs could play a great role. Recent studies prove that histone modifications may be also treated as targets in the treatment of depression. The examples show a significant effect of histone deacetylase inhibitors (HDACis) on the condition of MDD in clinical as well as in vivo studies. What is even more interesting is that the drugs currently administered to patients with depression, like selective serotonin reuptake inhibitors (SSRIs), were tested for their impact on PTMs, and the results have revealed significant post-treatment differences [20]. Herein, we present the results confirming the importance of PTMs in the diagnosis and treatment of depression.

### 3.1. Histone Modifications in the Diagnostics of Depression

#### 3.1.1. Animal Models of Depression

Due to the fact that MDD is an increasing problem in the modern society, it is worth expanding the diagnostic spectrum to better recognize the disease in patients. Furthermore, current diagnostic methods are based on questionnaires and surveys, which may not be sufficient to adequately determine the severity of the disease. Therefore, attention should be paid to PTMs, which are characteristic signs of depression. The epigenetic alterations have been studied mainly in animal models subjected to stress. An important question is whether the use of animal models in a research conducted on depression is effective. Although depression is a complex disease, symptoms such as anhedonia, behavioral despair, helplessness as well as changes in appetite and sleep patterns have been observed in animal models. These symptoms induced by models of stress form a depressive-like behavior.

In the case of studies performed on mouse models, we can observe that the pattern of PTMs depends on parts of the brain. In the research conducted on nucleus accumbens (NAc; a part of the reward circuitry) obtained from mice subjected to chronic stress one could observe: (i) decreased level of H3 acetylation, (ii) reduced levels of HDAC2 and 5, and (iii) reduced H3K14ac [62,63,64]. In addition to acetylation changes, there are methylation alterations in NAc. Here, methylation is regulated by G9a histone methyltransferase. Mice with genetically reduced G9a have an increased susceptibility to social defeat stress (SDS) [65]. Similarly to NAc, a decreased level of acetylation, i.e., H3K9ac, was observed in the hippocampus of mice subjected to SDS and decreased HDAC2 and 5 expression levels in the hippocampus after chronic restraint stress [66,67]. However, in the hippocampus, three weeks after the stress event there is a noticeable increase in acetylation in H4K12, an increase in HATs expression and a decrease in H3K4me3 [66]. As mentioned above, histone methylation exerts different effects, depending on the site of alteration. In this case, H3K4me3 is responsible for activating transcription [68]. Increased level of HATs and decreased level of HDACs are also observed in the cortex of mice after SDS [66]. Furthermore, SDS induces downregulation of *BDNF* and increases repressive histone methylation at *BDNF* promoters in the mouse hippocampus [69]. Nonetheless, adolescent social stress reduces H3K9me2 in the mouse medial prefrontal cortex [70]. Moreover, chronic stress also leads to a reduced level of histone crotonylation and an upregulation of chromodomain Y-like protein (CDYL; histone methyllysine reader, inhibits the activity of the nerve growth factor VGF and synaptic plasticity) in a medial prefrontal cortex, being accompanied by an elevation of anhedonia and social avoidance behaviors [60]. Another histone modification can be also associated with depression—histone3-lysine9-β-hydroxybutyrylation, which is triggered by β-hydroxybutyrate (can be used by the brain as a source of energy with an insufficiency of glucose). The level of this modification was decreased in the brains of stressed mice [71].

The stress factors may have different impacts on epigenetic changes in individuals with varying degrees of susceptibility to stress. The BALB mice strain (a concatenation of Bagg and Albino), in comparison to the C57BL/6, is characterized by reduced adaptability to stress factors. It is caused by the behavior of BALB dams, i.e., they provide a lower level of maternal care, additionally having different levels of gamma-aminobutyric acid (GABA) receptor and c-Fos expression [72,73]. Predictably, decreased H3 acetylation, increased HDAC2, and decreased activating H3K4me3 methylation in the region of the glial cell-derived neurotrophic factor (*Gdnf*; gene related to adaptation to chronic stress) promoters were observed in BALB mice. In C57BL/6 mice, only H3 acetylation was elevated and H3K27me3 decreased in the regions of *Gndf* promoters [74]. This methylation results in repressed transcription [68].

Especially interesting mice studies are those examining the effect of maternal separation on epigenetic alterations in offspring, due to introduction of depressive-like behavior. Interestingly, separation for a short period of time caused reduction in acetylation of H3 located in dopamine- and cAMP-regulated neuronal phosphoprotein (*DARPP32*) promoters, yet was not observed for long-term separation [75]. For middle-aged offspring, decreased expression of glucocorticoid receptor (GR) and decreased level of histone acetylation in the region of the *GR* promoters along with increased level of HDAC5 were observed. These changes became more pronounced in adult individuals [76]. It is curious that PTMs occur in a fetus when a mother is stressed and that the offspring of stressed pregnant mice show a depressive-like behavior. Furthermore, they have a decreased level of *BDNF* expression, an increased level of HDAC1 and HDAC2, and the decreased level of H3K14ac in the region of *BDNF* promoters in the hippocampus [63].

Studies conducted on rat models also present interesting results. In stressed rats, an elevated level of acetylation at H3, i.e., elevation of H3K18ac, can be observed in the medial prefrontal cortex [77], while in the hippocampus the level of H3 acetylation was decreased in the region of the *BDNF* promoters [50]. Moreover, the characteristic feature of stressed rats is an increased HDAC5 expression in the hippocampus and prefrontal cortex [78,79]. Changes in acetylation status can be observed in several parts of the brain: (i) decreased level of H3K14, H3K23, H4K16 acetylation in the prefrontal cortex [79], (ii) decreased level of H3K9, H3K14, H3K23, H4K16 and H4K12 acetylation in the hippocampus, (iii) decreased level of H4K8ac and increased level of H3K18ac and H3K12ac in the ventral hippocampus and (iv) increased level of acetylation in K9 and K14 of H3 and K5, K8, K12, K16 of H4 in the dorsal raphe [77,78].

Among the results of studies performed on rats, there are also noticeable changes in histone methylation. Rats exposed to chronic stress have decreased H3K9me3 in corticotrophin-releasing hormone receptor 1 (*Crhr1*) promoters [80]. Stress leads to changes in dentate gyrus and hippocampus, i.e., increases the level of H3K4me3, decreases the level of H3K27me3 and H3K9me1, which elevates with prolonged stress and decreases the H3K9me3 only in dentate gyrus [81]. The effect of the first two methylations remains unknown, and the last one results in repressed transcription.

Epigenetic changes were also studied in Indian house crows that were stressed by artificial lights at night. In their hippocampus reduced H3 acetylation and increased HDAC4 expression were found in the region of the *BDNF* promoters [82].

#### 3.1.2. Clinical Trials in Depression

PTM disturbances are characteristic for patients with MDD, and the results of studies with human participants are similar to these obtained in animal models. However, there are considerably fewer studies conducted on patients with depression than ones using animals. This is obvious due to the fact that patients’ brains are not collected in the standard procedures, and can only be obtained from suicide victims with their family’s consent. The obtained findings confirm elevated levels of HDAC2 and HDAC5 in the peripheral blood cells of depressive patients [83]. Furthermore, the increased level of HDAC5 is correlated with an increased expression of cyclic AMP-responsive element-binding protein 1 (CREB-binding protein), which is often a target in antidepressant therapy possessing histone acetyltransferase activity (to activate gene transcription) [84]. In the post-mortem studies, the methylation level in the prefrontal cortex of MDD patients was investigated. There was an increase in the transcriptionally repressing H3K9me3 in connexin 30 and 43 (CX30 and CX43; related to astrocyte communication) [85] and an increased level of transcriptionally activated H3K4me3 and an elevated synapsin-2 (SYN2) expression. Dysregulation of synapsin proteins is often associated with neuropsychiatric disorders [86]. The data collected in the above part of the review are presented in Table 3.

### 3.2. Histone Modifications in the Treatment of Depression

#### 3.2.1. Animal Models of Depression

Histone modifications can be used not only in the diagnosis of depression, but also as a therapeutic target. Often, medications that are used in the MDD therapy have a significant impact on the PTMs of histones.

In the studies carried out on mice models of depression, HDACis were the most frequently tested compounds. They inhibit the action of histone deacetylases, which results in an increased level of acetylation. The effect of elevation of histone acetylation facilitates gene transcription. The researchers confirmed that studied HDACis, crebinostat and neurinostat, inhibited the activity of HDAC1, 2, 3, 6 and 8 in *CREB*-reporter cells, and caused the elevation of acetylation of H3 and H4 in cultured mouse primary neurons. In addition, HDACis are able to reduce the level of depressive-like behavior. In stressed mice treated with belinostat, which is also an HDACi, a significant decrease in the level of immobility was observed in the tail suspension test [87,88,89]. Another well-known HDACi, sodium butyrate (NaB), was also found to have an antidepressant effect. In mice in which stress was induced with lipopolysaccharide (LPS) and in those with chronic restraint stress, it was observed that acute NaB administration reduced the depressive-like behavior [67,90]. NaB is also able to lower immobility scores in the tail suspension test and in the forced-swim test [91,92]. When it comes to a molecular analysis of mice brains, acute NaB administration increased H4 acetylation in the hippocampus, while chronic treatment decreased it [91]. In addition, systemic injection of NaB increased *BDNF* expression in the frontal cortex and hippocampus. Moreover, NaB increased H3 and H4 acetylation in the hippocampus and H3ac in the frontal cortex [67,92]. Considering another HDACi, MS-275, it also exerts an antidepressant effect. Infusion of this inhibitor into the hippocampus, amygdala or NAc results in a reduction of depressive-like behavior [64,93]. However, there is a study where chronic treatment with valproic acid (HDACi) did not show significant differences in the expression of HDAC2 and 5 or HDAC4, 6 and 8 in the mice leukocytes [83].

Due to the potential for using PTMs in the fight against depression, it is almost obvious to evaluate currently used antidepressants in the context of their effect on histone modifications. In studies with imipramine, a tricyclic antidepressant, it was confirmed that it reversed changes in H3 methylation induced by SDS in NAc in mice [94]. Additionally, chronic imipramine treatment resulted in decreasing histone methylation, an increase of histone acetylation at *BDNF* promoters and downregulation of HDAC5 in the hippocampus [69]. In contrast, in mice treated with fluoxetine (SSRI) an increased level of H4K12ac was observed [95].

When it comes to the studies conducted on rat models of depression, there are numerous pieces of evidence of the antidepressant effect of HDACi. NaB caused reduced immobility on the forced-swim test and the tail suspension test, and an increase in acetylation of H4 in the hippocampus of stressed rats [96,97]. MS-275 had a similar effect in rats as in mice, and when administered chronically, it showed a reduction in immobility in the forced-swim test and the tail suspension test. The antidepressant effect was accompanied by an increase in H3 acetylation and an elevated *CREB* and *BDNF* level in the ventrolateral orbital cortex of stressed rats [98]. Another example, vorinostat, reversed depressive-like behavior in rats with chronic alcohol consumption and raised their H3K9ac levels [99].

Fluoxetine, escitalopram and venlafaxine were among the SSRIs tested in rat models in order to evaluate whether they could improve depression severity levels. Rats subjected to chronic stress had an increase in H3K9me3 in dentate gyrus (DG) after fluoxetine treatment and an increase in H3 acetylation with escitalopram [81,100]. In the case of venlafaxine, it stopped the increase in HDAC5 expression and the decrease in acH3K9 that occurred after stress in rats [101].

Nevertheless, interesting studies on rat models examined, instead of the effect of chemical molecules on changes in histones, the relationship of PTMs in depression and treatment with methods such as acupuncture and electroconvulsive seizures. The former reversed the upregulation of HDAC2 in the hippocampus, which has appeared after chronic unpredicted mild stress in rats [102]. Rats that underwent electroconvulsive seizures had elevated levels of acetylation in H4 in the hippocampus within the *BDNF* and *CREB* promoters immediately after surgery, and two hours later the increase in H4 acetylation was accompanied by the decrease in acetylation in H3 [103].

A huge number of studies reflect a great interest in the area of PTMs in depression, and the above results demonstrate how important the relationship between histone modification and depression pathophysiology is.

#### 3.2.2. Clinical Trials in Depression

Unfortunately, there are still few clinical trials investigating the link between PTMs and depression. So far only two studies focused on evaluating the impact of SSRIs. Each study was conducted in a group of patients with diagnosed MDD. Citalopram from the SSRI group was used in a group of 25 patients and its effect on histone methylation was examined. A decrease in H3K27me3 in blood was observed as a result of the treatment and a negative correlation between the methylation and the depression severity was shown. This confirms the relationship of histone modification with depression that was discovered in animal models [104].

A group of 48 patients was treated with other drugs belonging to SSRIs, fluoxetine or paroxetine (NCT01182103). The treatment caused a slight increase in the level of acetylation in H3 and H4 within the *BDNF* promoters. This allows to suggest that the use of histone modifications as a therapeutic target has a great potential.

Interestingly, 234 patients were treated (NCT01912196) with S-adenosyl methionine (MSI-195), which is the methyl-donating substrate of histone methyltransferases. The drug was given alongside ongoing antidepressant medication. Although no significant differences in the effect of this drug were found in relation to the depression severity scales (HamD17, MADRS, IDS-SR30) in comparison to the placebo group [105], the authors indicated that the test scores were better in the tested group than the placebo group in the first phase of the study. Moreover, the group from the second phase was demographically and clinically different, which could have contributed to the general lack of significant statistical differences. This confirms that there is a need for an individual approach for patients with MDD and that current treatment is insufficient and should be extended in order to help anyone with depression.

The data presented in this part of review are totaled in Table 4.

## 4. miRNA

Epigenetics associated with small non-coding RNAs are most spectacularly developed in the context of miRNAs, while a large body of evidence suggests an impact of their interference on a plethora of biological phenomena [106]. They are short, about 21-nucleotide long, single-stranded RNAs that use mainly two mechanisms of post-transcriptional gene silencing (PTGS). Namely, they either cause degradation of the transcript or disrupt the process of translation, both of which are placed in cytoplasm. However, recently it was found that miRNAs are also present in the nucleus, where they are involved in both repression and activation of transcription [10]. To perform their tasks, they need to undergo a multistep process of maturation, initialized with a transcription of long primary miRNAs (pri-miRNAs) by RNA polymerases II or III. Then, pri-miRNAs are shortened to 70-nuclotide long precursor miRNAs (pre-miRNAs) by a Microprocessor complex consisting of RNase III enzyme Drosha and a double-stranded RNA-binding protein, Di George syndrome critical region 8 gene (DGCR8), followed by creation of double-stranded miRNA duplexes (ds-miRNAs) by additional activity of Dicer. Aside from Dicer, ds-miRNAs associate with Argonaute (Ago) and trans-activation response RNA-binding protein (TRBP) forming the RNA-inducing silencing complex (RISC) in cytoplasm, while the final step involves degradation of one of the RNA strands to form a working miRISC complex. Interestingly, the complex can also be constituted of only Ago and miRNA molecule; then it is called minimal RISC complex. The canonical mechanism of RNA interference in cytoplasm is well-established and is achieved by creating Watson–Crick hydrogen bonds between positions from 2 to 8 of miRNA 5′ end (the so-called seed region) and miRNA response element (MRE) located in most cases in 3′ untranslated region (3′-UTR) and rarely in 5′-UTR or coding region of transcript [107,108,109,110]. However, in the case of ~15–80% of miRNAs, these interactions can be established through non-canonical mechanisms [111,112]. While their role in the nucleus is not fully understood yet, two main mechanisms have been established, i.e., PTGS mediated by nuclear RISC for miRNAs and other endonuclear non-coding RNAs and direct interactions with the promoters, where the resulting down- or upregulation depends on location and methylation status of target sides [113,114]. For the latter, three mechanisms have been proposed: (i) RNA–RNA model, where direct interaction of minimal RISC with the transcript resulting from recruitment of histone modifiers [115,116,117,118], (ii) RNA–DNA hybrid model, in which Ago is guided by miRNA to the TATA box or binding sites of transcription factors (TFs) triggering recruitment of histone modifiers or TFs [119,120,121,122,123] and (iii) RNA–DNA triplex model, where topology of DNA is changed due to binding of miRNA to major groove using Hoogsteen or reverse Hoogsteen bonds, which recruit TFs [124,125].

Through those mechanisms one miRNA molecule can influence expression of hundreds of target genes, thus, together they have an enormous impact on transcriptome and proteome [10]. It is then not surprising that changes of their expression have been found to be involved in etiopathogenesis of many disorders, including cancers, diabetes [126], neurodegenerations and neuropsychiatric ones [127,128,129]. This is also true for depression, where in the last decade several dozen miRNAs have been found to be deregulated. However, there is a debate surrounding the consistency of the results obtained in different studies [130]. The establishment of such a panel of peripheral biomarkers is especially important, since the collection of a blood sample is relatively harmless for the patient and the detection of miRNA in plasma or serum is not challenging [131].

### 4.1. miRNAs and the Brain—A Link between Neuroplasticity and Depression

It has been found that miRNAs are highly expressed in brain tissue, where they play multiple roles, mostly in nervous system development, but also in the regulation of neuronal and synaptic plasticity [132,133,134,135]. Not surprisingly, disturbances in brain plasticity were also found to be involved in the pathogenesis of depression [7,136]. Several studies done on post-mortem brains of depressed patients or animal models of depression showed reduction of the prefrontal cortex (PFC) and hippocampus volumes, neuronal atrophy, decreased length and branching of dendrites, etc. [137,138,139,140]. Interestingly, antidepressant treatment seems to reverse these processes, since in preclinical studies using stressed-induced depression-like behavior, chronic, but not acute, administration of antidepressants caused neurogenesis in the hippocampus, which was further confirmed in post-mortem hippocampal cells of treated depressed patients [141,142,143,144,145]. Here, the working hypothesis is that disturbances of neuroplasticity affect homeostatic control of synaptic connections and the ability of individuals to cope with stressful life events, thus causing depression to emerge [146].

Indirect evidence of miRNA involvement in synaptic plasticity was gathered using Dicer knockout mice. These animals showed microcephaly, reduced dendritic branch elaboration, increased length of dendritic spine but without increase of spine density, while specific to only Purkinje cells, knockout resulted in the death of those cells and ataxia [147,148]. Furthermore, in another study, a knockout of DGCR8 affected cognitive performance and caused neuronal deficiency in mice [149]. All those results indicated the importance of miRNAs in proper functioning of nervous tissue. Indeed, up to date, several candidates have been proposed, i.e., miR-9, miR-16, miR-124, miR-125b, miR-132, miR-134 and miR-138 [131]. Some of them are tightly linked, i.e., they regulate or are regulated by proteins involved in neuroplasticity—BDNF, CREB (cAMP response element-binding protein), and SIRT1 (Sirtuin 1). Among these proteins, BDNF is one of the key factors that play a pivotal role in neuroplasticity. Moreover, it is also crucial in neuroinflammation, thus making it the perfect candidate that can bridge both neurogenic and inflammatory hypotheses of depression [150].

### 4.2. Circulating miRNAs as Biomarkers of Depression

#### 4.2.1. Blood, Plasma and Serum miRNAs as Biomarkers of Depression

As aforementioned, the use of circulating miRNA molecules as peripheral markers seems tempting, especially considering the ease of collecting the material and performing tests. Furthermore, the diagnostic potential of individual serum miRNAs, e.g., miR-34a-5p, let-7d-3p, miR-221-3p, miR-451-a, was evaluated as high via receiver operating characteristic (ROC) curve analyses [151]. The same statistical approach also indicated that rather than single molecules, a panel of miRNAs (e.g., miR-4743, -26b, -1972, -4498, -4485 examined in PBMCs) could serve as a tool for MDD diagnosis [152]. However, a recent review by Yuan et al. found that there is little replication across the studies [130]. Authors identified 23 clinical studies and six performed using the animal models, which discovered 178 dysregulated miRNAs. Unfortunately, results for only two of them were replicated, i.e., miR-132 in four studies and miR-16 in two preclinical studies. Interestingly, the former molecule is tightly connected to neuroplasticity through BDNF [153], while the latter is involved in the regulation of serotonin transporter (SERT) and is thought to be implicated in the mechanism of fluoxetine, belonging to the SSRI antidepressant class, where it mediates neurogenesis in the hippocampus [154,155]. Moreover, miR-16 was found to be reduced in depressed patients’ cerebrospinal fluid (CSF), but not in blood, and when rats were treated with anti-miR-16 they exhibited depression-like behaviors, as well as increased levels of CSF serotonin and raphe nuclei SERT [156]. Even more interestingly, rats resilient to the CNS procedure had elevated serum levels of miR-16 [157]. This could indicate a link between circulating miRNAs and the ones present in the CNS. However, such regularity has not been yet observed in clinical studies and the mechanism of such a relationship remains elusive. Nevertheless, Yuan et al. placed guidelines in their review for the future research in this topic to improve replicability of the results from study to study [130].

#### 4.2.2. Cerebrospinal Fluid miRNA as a Biomarker of Depression

Although collection of CSF is not as easy as collection of blood, it is thought to better represent fluctuations in miRNAs status than peripheral tissue or even post-mortem brain samples [158,159]. Its close interaction with the brain makes CSF an ideal candidate for study changes in the CNS [160,161]. Moreover, CSF-derived markers should have superior sensitivity, specifically when compared to blood ones, as it was shown in the meta-analysis of CNS cancers [162]. This statement seems to be also true for depression, since in the study mentioned earlier expression of miR-16 in CSF, but not in blood, was lower in depressed patients when compared to healthy subjects and was positively correlated with the amount of serotonin [156]. Another report that compared serum and CSF miRNAs in depressed patients found that from 16 deregulated miRNAs in CSF only four were also deregulated in serum [151]. Although these results are promising, there is too little research to predict whether CSF-derived miRNAs could be superior markers of the disease.

### 4.3. miRNAs and Depression Therapy

Treatment of the depression seems to be especially challenging. Although many substances with antidepressant properties have been developed over the years and a plethora of drugs is now available, still about 1/3 of the patients do not respond to traditional pharmacotherapy [163]. Moreover, proper evolution of the patients’ response to treatment can be performed only after six weeks of therapy. It is therefore not surprising that markers are sought to enable an earlier evaluation of the effectiveness of therapy. Since miRNA concentration can rapidly change in order to compensate for changes in environment [164], they seem to be perfect candidates for such markers [165]. Indeed, a great body of evidence was provided by clinical, preclinical and in vitro studies showing that changes of miRNA expression can reflect the progress of depression treatment (Table 5) [159].

**Table 5:** Summary of studies reporting evidence of miRNA involvement in treatment response.

Treatment	Model/Patients	Type of Biological Material	Results/Findings	Paper
**Selective serotonin reuptake inhibitors (SSRIs)**				
Fluoxetine	Mice infused with antidepressant into raphe nuclei	Raphe nuclei	Fluoxetine infusion caused overexpression of **miR-16**, which resulted in reduction of SERT expression.	[154]
	MS in Sprague Dawley rat	Hippocampi	MS reduced expression of **miR-451**, while fluoxetine treatment reversed this change.	[166]
	SK-N-SH and SH-SY5Y neuroblastoma cell lines	Cell lysates	Fluoxetine increased expression of **miR-572** and **miR-663a**, which are involved in fundamental neurodevelopmental processes.	[167]
	Genetically modified mouse models, expressing higher or lower levels of **miR135**; chronic social defeat model	Raphe nuclei	Mice overexpressing **miR-135** were resilient to social defeat, while knockout induced anxiety-like behaviors and decreased the response to antidepressants.	[168]
	PTSD in mice	Prefrontal cortices	Reduction of **miR-1971** expression after administration of fluoxetine.	[169]
Paroxetine	1C11 neuroectodermal cell line	Cell lysates	**miR-16** overexpression resulted in reduction of SERT translation and [^3^H]-paroxetine binding sides.	[154]
	U87 human glioblastoma–astrocytoma cell line	Cell lysates	Treatment with paroxetine induced expression of BDNF and was limited by upregulation of **miR-30a-5p**.	[170]
	80 human LCLs form healthy adult females	Cell lysates	Paroxetine-sensitive LCLs overexpressed **miR-151-3p**.	[171]
	84 depressed patients and 78 control volunteers	Serum	Eight-week treatment resulted in increased **miRNA-451a** levels, decreased **miRNA-34a-5p** and **miRNA-221-3p** levels and reduced HAM-D scores.	[172]
Citalopram	32 depressed patients, classified into remitters and non–responders, based on changes in HAM-D scores, and 18 controls	Blood	Downregulation of **miR-1202** at baseline was found in remitters, when compared to non-responders and controls; eight-week citalopram administration upregulated **miR-1202** only in remitters.	[173]
	45 untreated depressed patients, 32 treated with citalopram and 32 healthy volunteers	Plasma	**miR-132** was upregulated in untreated depressed patients, when compared to healthy controls and citalopram-treated patients; and its expression decreased after two-month citalopram administration, while at the same time **miR-124** expression increased.	[174]
Escitalopram	MS and CUS in Sprague Dawley rats	NAc and striatum	Escitalopram normalized increased expression of **miR-326** after MS and CUS.	[175]
	10 depressed patients	Blood	Treatment significantly altered expression of 30 miRNAs: 28 were upregulated, while two were downregulated.	[176]
	158 depressed patients	Blood	Downregulation of **miR-146a-5p**, **miR-146b-5p** and **miR-24-3p** after eight-week treatment with escitalopram	[177]
**Serotonin–norepinephrine reuptake inhibitors (SNRIs)**				
Duloxetine	Kunming mice in CUMS	Hippocampi and frontal lobes	Three-week administration of duloxetine caused increased expression of **miR-18a**, and **miR-132** in hippocampus, while **miR-134** and **miR-124a** were downregulated; only **miR-18** was upregulated in frontal lobe.	[178]
	258 depressed patients	Blood	16 miRNAs changed specifically according to duloxetine treatment, while **miR-146a-5p**, **miR-146b-5p**, **miR-24-3p**, **miR-425-3p** and **miR-3074-5p** were all downregulated and strongly correlated.	[177]
Desvenlafaxine	20 depressed patients	Blood	Using MRI **miR-1202** levels were correlated with changes in brain activity and connectivity occurring during an eight-week antidepressant treatment.	[179]
**Miscellaneous**				
Imipramine (TCA)	Mice in CSDS	Blood	Administration of imipramine downregulated **miR-146b-5p**, **miR-24-3p** and **miR-425**, while no differences were detected in the non-responders or saline control groups.	[177]
Ketamine	MS in Sprague Dawley rats	Hippocampi	Upregulation of **miR-598-5p** after treatment.	[166]
	Sprague Dawley rats	Hippocampi	Three injections of ketamine once per day caused downregulation of **miR-206**, while increase of BDNF level.	[180]
ECT	MS in Sprague Dawley rats	Hippocampi	Upregulation of **miR-598-5p** after treatment.	[166]
	ECS in Sprague Dawley rats	Blood and brains	Chronic and acute ECS upregulated **miR-212** in dentate gyrus, while only chronic ECS increased level of this molecule in blood.	[181]
	37 depressed patients and 34 healthy controls	Blood	Elevated levels of **miR-126-3p** and **miR-106a-5p** were normalized after ECT.	[182]
	24 TRD depressed patients and 20 healthy controls	Blood	**miR-let-7b** and **miR-let-7c** were downregulated after treatment as compared to the controls.	[183]
CBT	24 depressed patients	Blood	Significantly higher levels of **miR-135a** after 12 weeks of CTB, when compared to the same patients before therapy and with population treated with SSRIs.	[168]
Different antidepressants	68 patients treated with escitalopram (SSRI) or nortriptyline (TCA)	Blood	Downregulation of **miR-146a-5p**, **miR-146b-5p**, **miR-24-3p** and **miR-425-3p** after treatment.	[177]
	32 depressed patients	PBMCs	Significantly increased level of **miR-124** in depressed patients as compared to the healthy controls being decreased after eight-week antidepressant treatment.	[184]

SERT—serotonin transporter; MS—maternal separation; LCLs—human lymphoblastoid cell lines; CBT—cognitive behavioral therapy; CUS—chronic unpredictable stress; NAc—nucleus accumbens; CUMS—chronic unpredictable mild stress; MRI—magnetic resonance imaging; CSDS—chronic social defeat stress; PBMCs—peripheral blood mononuclear cells; TRD—treatment-resistant depression; HAM-D—24-item Hamilton Depression Scale; ECS—electroconvulsive stimulation.

#### 4.3.1. SSRI

In an earlier mentioned study, fluoxetine increased miR-16 levels in serotonergic raphe nuclei, which resulted in downregulation of SERT [154]. Moreover, the same antidepressant was shown to upregulate two miRNAs, i.e., miR-451 and miR-598-5p, that were repressed in the hippocampus of mice that underwent maternal separation (MS) procedure [166]. Further evidence was introduced by in vitro studies, where two neuroblastoma cell lines, i.e., SK-NSH and SH-SY5Y, showed consistent overexpression of miR-572 and miR-663a after treatment with fluoxetine [167]. Another antidepressant belonging to the SSRI class, paroxetine, was also studied in vitro, where in human glioblastoma–astrocytoma cell line U87 it increased expression of BDNF and its transcriptional inhibitor miR-30a-5p [170]. Moreover, overexpression of miR-151-3p was found in human lymphoblastoid cell lines (LCLs), which were sensitive to this antidepressant [171]. Even more interestingly, patients that responded to the treatment with paroxetine exhibited increased miR-451a and decreased miR-34a-5p and miR-221-3p serum expression, while these molecules were correlated with the severity of the disease measured by the 24-item Hamilton Depression Scale (HAM-D) [172]. Similar results were obtained for blood levels of miR-1202, when treatment-naïve patients were treated with citalopram [173]. After the therapy using this SSRI class antidepressant, they were divided into two groups: remitters (REMs) and non-responders (NRES) using HAM-D scores, and it was revealed that: (i) the former group had lower expression than NRES and control groups, (ii) no differences were found between the latter group and healthy individuals, and (iii) negative correlation was present between change in depression severity and expression of miR-1202. In another clinical study, it was shown that citalopram induced changes in serum expression of two miRNAs, i.e., miR-132 was upregulated, while miR-124 was downregulated [174]. Particularly interesting results were also found in relation to (*S*)-stereoisomer of citalopram—escitalopram. It was revealed that chronic unpredictable stress (CUMS), which induces depression-like behaviors in rats, caused upregulation of miR-326 in the nucleus accumbens and its downregulation in the striatum, while administration of escitalopram normalized it [175]. On the other hand, the antidepressant had no effect on another deregulated molecule—miR-9. Impressively, in depressed patients, 12-week escitalopram administration changed the expression of 30 miRNAs, and after target gene prediction and functional annotation analysis, they were revealed to be involved in pathways associated with neuronal brain function [176].

#### 4.3.2. SNRIs and TCA

Another class of antidepressants—SNRIs—were also found to influence miRNA expression. One of them, desvenlafaxine, similarly to citalopram, influenced expression of miR-1202 in depressed patients [179]. Authors using functional magnetic resonance imaging (fMRI) and spectroscopy to detect glutamate showed that the level of this molecule was correlated with brain activity and connectivity throughout the treatment, and since miR-1202 was also correlated with glutamate, it was speculated that its mechanism of modulating antidepressant treatment outcome could involve changes in the glutamatergic system. Another SNRI, duloxetine, was found to upregulate miR-132 in the hippocampus and miR-18a in the hippocampus and frontal lobe, while downregulating hippocampal expression of miR-134 and miR-124a in mice exposed to CUMS [178]. This could indicate that this antidepressant targets primarily the hippocampus, and to a less extent the frontal lobe, where it deregulates miRNA expression. Duloxetine was also investigated in a clinical study, where it downregulated miR-146a-5p, miR-146b-5p, miR-24-3p and miR-425-3p in 516 samples of peripheral blood from depressed patients after antidepressant treatment vs. the same patients before treatment [177]. Moreover, authors replicated the results in two independent cohorts (apart from miR-425-3p, which was not downregulated in the second cohort) treated with either escitalopram or nortriptyline, which is a tricyclic antidepressant (TCA). Even more compelling, in the same paper three out of four miRNAs, i.e., miR-146b-5p, miR-24-3p and miR-425, were also downregulated when peripheral blood samples of mice exposed to the chronic social defeat stress (CSDS) before and after administration of imipramine belonging to the TCAs were compared. Lastly, these molecules were upregulated in post-mortem samples of the ventrolateral prefrontal cortex (vPFC) of suicidal depressed individuals when compared to psychiatrically healthy controls. Interestingly, these miRNAs are involved in regulating *MAPK* and *Wnt* signaling pathways, which link them to, among others, BDNF [177]. Another two clinical studies that used more than one antidepressant showed changes in miRNA in the peripheral blood of treated patients [184,185]. However, the set of altered miRNAs differed between studies. Since these three studies were conducted on different ethnic groups, i.e., populations of Canada, China and France, it could indicate that the ethnicity of the patients plays a major role in the changes of miRNA expression and must be taken into consideration in future studies.

## 5. lncRNA in Depression

### 5.1. lncRNA Description and Functions

Long noncoding RNAs belong to the least known group of noncoding RNA molecules, probably as a result of their low expression level and poor sequence conservation [186,187,188]. However, they have gained the attention of scientists due to their capability of gene expression regulation. lncRNAs, above 200-nucleotide-long RNA particles, do not encode any proteins. They are multitask molecules participating in the regulation of DNA methylation, histone modifications and chromatin remodeling, as well as controlling gene expression via transcriptional and post-transcriptional mechanisms [189,190]. lncRNAs are known to interact with mRNAs, serve as miRNA sponges, decoy or scaffold molecules [187,191]. Therefore, lncRNAs may be present in both nucleus and cytoplasm [192].

There are several ways of classifying lncRNAs. Taking into account their origin, one could distinguish intergenic and intronic lncRNAs. Intergenic lncRNAs are transcribed from intergenic–noncoding regions between genes, while intronic ones are obtained from introns of protein-coding genes [193]. To date, intergenic lncRNAs also known as lincRNAs, are some of the best described representatives of lncRNAs [194]. Moreover, lncRNAs can be transcribed in either sense or antisense orientation [195]. Considering the regulatory function of lncRNAs, they may act in two ways: cis—interacting with neighboring protein-coding genes, and trans—influencing expression of distant genes [196]. Finally, the length also makes them simply categorized into small (<950 nt, the most abundant group), medium (950–4800 nt) and large (>4800 nt) lncRNAs [194].

### 5.2. lncRNA in the Nervous System

Interestingly, a significant part of known lncRNAs has been found to be expressed in brain tissue [197,198]. There are many described lncRNAs (Table 6) that are involved in different stages of brain development and its activity. Some important examples are RMST, which is engaged in neurogenesis, or MALAT1, which participates in synaptogenesis [195,199,200]. Therefore, studies have started to investigate the roles of lncRNAs in the pathogenesis of mental disorders, including depression [189].

Although the research is in its infancy, several lncRNAs correlated with MDD have been already reported. The study of Hosseini et al. demonstrated that lncRNAs *NRON* (repressor of nuclear factor of activated T cells NFAT) and *BACE1-AS* (an antisense transcript of B-secretase 1) modulate expression of relevant protein-coding genes involved in proper nervous system function. Furthermore, downregulation or knockdown of BDNF-AS lncRNA upregulated BDNF expression, leading to elevation of GDNF and EPH receptor B2 and activation of neuron development [133]. BDNF-AS was also found in animals showing the ability to halt BDNF transcription [201].

Another research group [202] performed microarray-based genome-wide studies and identified six lncRNAs: TCONS_00019174, ENST00000566208, NONHSAG045500, ENST00000517573, NONHSAT034045, and NONHSAT142707, which exhibited significant expression changes in patients with depression as compared to patients after remission. The group also showed that the lncRNAs have significant correlation with suicide risk [202]. The study by Liu et al. [201] reported that overexpression of NONHSAG045500 inhibits transcription of serotonin transporter SERT in vitro.

The animal model with depression-like symptoms triggered by the procedure of learned helplessness proved the previous results, that lncRNAs may have an impact on regulatory mechanisms implicated in depression [200] This study suggested that disturbance of lncRNAs in the brain may be involved in stress-induced pathologies such as synaptic dysfunction. Other studies with mice revealed that depression-related behavioral tests, such as elevated plus maze, forced-swim test, and tail suspension test themselves could cause the changes of mRNA and lncRNA expression [203]. However, the general translation of animal studies might cause some difficulties due to low conservation of lncRNAs between humans and other species [188,197].

### 5.3. lncRNAs as Depression Biomarkers

Similar to other non-coding RNAs, miRNAs and lncRNAs are expected to become MDD biomarkers [189,204]. Despite the evident regulatory role of lncRNAs in expression of essential genes for neural system development, a number of studies show that lncRNA expression may vary in several neuropsychiatric disorders, such as autism spectrum disorders, schizophrenia and depression [199]. To the best of our knowledge, there is only one study, performed by Liu et al., showing 2007 diversely expressed lncRNAs in the peripheral blood of patients with depression as compared to the control group. Also, attempts have been made to analyze co-expression of lncRNAs and mRNAs, giving a broader view on epigenetic regulation in depression. From those 2007 lncRNAs: chr10:874695-874794, chr10:75873456-75873642, and chr3:47048304-47048512 were found to interact with mRNAs differentially expressed in depressed patients [205]. Thus, lncRNAs may be involved in the pathogenesis of depression also via mRNA regulation [189]. However, only a few studies have examined circulating lncRNAs in the blood of patients with depression [190,202,205,206,207]

Currently, there are several ongoing pre-clinical trials evaluating the potential of using lncRNAs as disease biomarkers [188]. For instance, GAS5 lncRNA may serve as a possible biomarker for type 2 diabetes (T2DM) and coronary artery disease (CAD) [208]. Moreover, NBAT-1 and SNGH1 may become diagnostic markers of tumorigenesis in various cancer types [209,210]. To date, no clinical approaches in depression treatment have been published.

### 5.4. Future Research

To date, the knowledge regarding lncRNAs in depression and its therapy is rather scarce. As some lncRNAs have been indicated as especially interesting in other psychiatric disorders, e.g., Gomafu lncRNA related to schizophrenia or AS-Uchl1 in Parkinson’s disease, it would be interesting to evaluate their contribution to depression pathogenesis and treatment efficacy in numerous novel studies [198,199]. As mentioned earlier, lncRNA NONHSAG045500 shows an interesting correlation with serotonin transporter, making it a promising target for future antidepressant therapy. Even though there are only several reports about lncRNAs’ role in depression, the lncRNA abundance in the brain leaves a wide margin for prospective research.

## 6. Conclusions

Collected data strongly suggest that epigenetic modifications are associated with etiopathogenesis of depression, neuroplasticity, antidepressant treatment and, finally, could be regarded as potential depression biomarkers. Interestingly, epigenetic marks are ones introduced during harmful early-life events, reflecting an increased vulnerability to depression during one’s lifetime. Presented data also suggest that, from the clinical point of view, HDACis could possibly serve as new antidepressant agents even in the near future. Moreover, while the involvement of DNA methylation, histone modifications and miRNA expression changes have been substantially investigated in the pathogenesis and therapy of depression, the research oriented toward lncRNAs is still in its infancy. Therefore, studying interactions among lncRNAs, miRNAs and numerous target mRNAs could be an interesting direction of future research, not only in the context of depression, but also other neuropsychiatric diseases. Similarly, there is still a lack of data providing a global view on the crosstalk among the above epigenetic marks in depression. However, conclusions from currently available studies are often inconsistent, being possibly associated with methodological issues or ones emerging from ethnic differences. Having obtained promising results so far, we believe there is still a great need for further research addressing these important issues and showing a broader picture of the epigenetic landscape in depression and its therapy.

## Figures and Tables

**Table 3:** Studies reporting epigenetic alterations in the course of depression.

Histone Modification	Material	Model of Depression/Patients	Results	Paper
H3K12ac	Ventral hippocampus	Chronic social defeat stress in rats	Increased level of acetylation	[77]
H3K14ac	Nucleus accumbens	Social defeat stress in mice	Decreased level of acetylation	[64]
	Hippocampus	Gestational stress in offspring of pregnant mice	Increased level of acetylation	[63]
	Prefrontal cortex	Chronic unpredicted stress in rats	Decreased level of acetylation	[79]
	Dorsal raphe	Chronic social defeat stress in rats	Increased level of acetylation	[77]
	Hippocampus	Chronic unpredicted stress in rats	Decreased level of acetylation	[79]
H3K18ac	Medial prefrontal cortex	Chronic social defeat stress in rats	Increased level of acetylation	[77]
	Ventral hippocampus	Chronic social defeat stress in rats	Increased level of acetylation	[77]
H3K23ac	Prefrontal cortex	Chronic unpredicted stress in rats	Decreased level of acetylation	[79]
	Hippocampus	Chronic unpredicted stress in rats	Decreased level of acetylation	[79]
H3K27me3	Ventral striatum	Chronic stress in C57BL/6 mice	Increased level of methylation	[74]
	Hippocampus	Acute and restraint stress in rats	Decreased level of methylation	[81]
	Dentate gyrus	Acute and restraint stress in rats	Decreased level of methylation	[81]
H3K4me3	Hippocampus	Social defeat stress in mice	Decreased level of methylation	[66]
	Ventral striatum	Chronic stress in BALB mice	Decreased level of methylation	[74]
	Hippocampus	Restraint stress in rats	Decreased level of methylation	[81]
	Prefrontal cortex	MDD suicide victims	Increased level of methylation	[86]
H3K9ac	Hippocampus	Social defeat stress in mice	Decreased level of acetylation	[66]
	Hippocampus	Chronic unpredicted stress in rats	Decreased level of acetylation	[78]
	Dorsal raphe	Chronic social defeat stress in rats	Increased level of acetylation	[77]
H3K9me1	Hippocampus	Acute stress in rats	Decreased level of methylation	[81]
	Dentate gyrus	Acute stress in rats	Decreased level of methylation	[81]
H3K9me2	Medial prefrontal cortex	Adolescent social stress in mice	Decreased level of methylation	[70]
H3K9me3	Hypothalamus	Chronic unpredictable mild stress in rats	Decreased level of methylation	[80]
	Dentate gyrus	Chronic restraint stress in rats	Decreased level of methylation	[81]
	Prefrontal cortex	MDD suicide victims	Increased level of methylation	[85]
H4K12ac	Hippocampus	Social defeat stress in mice	Increased level of acetylation	[66]
	Ventral hippocampus	Chronic social defeat stress in rats	Decreased level of acetylation	[77]
	Hippocampus	Chronic unpredicted stress in rats	Decreased level of acetylation	[78]
	Dorsal raphe	Chronic social defeat stress in rats	Increased level of acetylation	[77]
H4K16ac	Prefrontal cortex	Chronic unpredicted stress in rats	Decreased level of acetylation	[79]
	Hippocampus	Chronic unpredicted stress in rats	Decreased level of acetylation	[79]
	Dorsal raphe	Chronic social defeat stress in rats	Increased level of acetylation	[77]
H4K5ac	Dorsal raphe	Chronic social defeat stress in rats	Increased level of acetylation	[77]
H4K8ac	Ventral hippocampus	Chronic social defeat stress in rats	Decreased level of acetylation	[77]
	Dorsal raphe	Chronic social defeat stress in rats	Increased level of acetylation	[77]

ac—acetylation, MDD—major depressive disorder, me—methylation.

**Table 4:** Studies reporting the impact of treatment on histone modifications and histone deacetylases (HDAC) in depression.

Treatment	Material	Model of Depression/Patients	Results	Paper
Histone deacetylase inhibitors				
Crebinostat	Primary neurons	Mice	Inhibition of the activity of HDAC1, 2, 3, 6 and 8 and elevation of H3ac and H4ac	[87]
Ms-275	Ventrolateral orbital cortex	Social defeat stress in rats	Increase in H3ac	[98]
Neurinostat	Primary neurons	Mice	Inhibition of the activity of HDAC1, 2, 3	[88]
Sodium butyrate	Hippocampus	Acute or chronic stress in mice	Increase in H4ac after acute administration, but decrease after chronic treatment	[91]
	Hippocampus and frontal cortex	Chronic restraint stress in mice	Increase in H3ac and H4ac in the hippocampus and H3ac in the frontal cortex	[92]
	Hippocampus	Chronic variable stress in rats	Increase in H4K12ac	[96]
Selective serotonin reuptake inhibitors				
Citalopram	Blood	MDD patients	Decrease in H3K27me3	[104]
Escitalopram	Hippocampus	Maternal separation in rats	Increase in H3ac	[100]
Fluoxetine	Forebrain neocortex	Infant maternal separation in mice	Increase in H4K12ac	[95]
	Dentate gyrus	Chronic restraint stress in rats	Increase in H3K9me3	[81]
Venlafaxine	Hippocampus	Chronic unpredicted stress in rats	Inhibition of HDAC5 expression, increase and decrease in H3K9ac	[101]
Tricyclic antidepressants				
Imipramine	Nucleus accumbens	Social defeat in mice	Reversed changes in H3me triggered by stress	[94]
	Hippocampus	Chronic social defeat stress in mice	Decrease in H3K4me2, increase in histone acetylation at BDNF promoters and downregulation of HDAC5	[69]

ac—acetylation, HDAC—histone deacetylase, MDD—major depressive disorder, me—methylation.

**Table 6:** Examples of lncRNA roles in neural development and pathogenesis.

lncRNA	Mechanism of Action	Function	Pathological State	References
BC200	Inhibition of translation initiation in dendrites	Support of long-term synaptic plasticity	Alzheimer’s disease and Parkinson’s disease	[188,195]
UCHL1-AS	Enhancement of translation of UCHL1 mRNA	Involved in proper neurons development	Parkinson’s disease	[188]
HTT-AS	Negative regulation of HTT expression	Maintenance of proper nervous system functioning	Huntington’s disease	[188]
Camkk1	Regulation of Ca2+/calmodulin-dependent protein kinase II	Long-term memory maintenance	Lack of evidence	[195,199]
Evx1as and Hox5b/6as are	Interaction with H3K4 histone and histone methyltransferase	Maintenance of proper nervous system functioning	Lack of evidence	[199]
Tuna	Regulation of gene expression in neural cells	Maintenance of proper nervous system functioning	Lack of evidence	[199]
Sox2OT	Overlapping with Sox2 gene	Involved in development of neural stem and progenitor cells	Alzhaimer’s disease and Parkinson’s disease	[195,199]
RMST	Regulation of neural cells differentiation	Involved in neurogenesis	Lack of evidence	[199]
Nkx2.2AS	Regulation of Nkx2.2 mRNA level	Maintenance of oligodendrocytes differentiation	Lack of evidence	[195,199]
HAR1F	Regulation of migration of neural cells and forebrain organization	Development of human embryonic neocortex	Huntington’s disease	[195,199]
Malat1	Regulation of SR-family splicing factors	Involved in synaptogenesis	Lack of evidence	[199]
BDNF-AS	Reducing level of BDNF factor	Involved in neurons development	Schizophrenia	[195,199]
Gomafu	Binding to splicing factors	Involved in neural development	Schizophrenia	[195,199]
